# CAZome comparison in relation to host plant for selected *Sordariomycete* and *Dothidiomycete* plant pathogenic fungi

**DOI:** 10.3389/ffunb.2026.1789997

**Published:** 2026-03-10

**Authors:** Hazal Kandemir, Mao Peng, Max Koster, Johannes Z. Groenewald, Pedro W. Crous, Andrei S. Steindorff, Ronald P. de Vries

**Affiliations:** 1 Evolutionary Phytopathology, Westerdijk Fungal Biodiversity Institute, Utrecht, Netherlands; 2 Fungal Physiology, Westerdijk Fungal Biodiversity Institute, Utrecht, Netherlands; 3 U. S. Department of Energy Joint Genome Institute, Lawrence Berkeley National Laboratory, Berkeley, CA, United States

**Keywords:** CAZy, dothidiomycetes, plant pathogenic fungi, plant polysaccharide degradation, sordariomycetes

## Abstract

**Introduction:**

While most studies focus on the effectors involved in plant infection, another important aspect is the degradation of the plant cell wall, as this is the main physical barrier protecting the plant from pathogens. The plant cell wall mainly consists of polysaccharides, proteins and the aromatic polymer lignin, but the type of polysaccharide differs significantly between plant types, species and tissues. It can therefore be expected that pathogens of specific plants have evolved to produce those plant polysaccharide degrading enzymes that match the polysaccharides in the cell wall of their host plant.

**Methods:**

In this study, we compared the plant polysaccharide degradation potential of 56 *Dothideomycetes* and 42 *Sordariomycetes* species to identify evolutionary patterns related to either host plant or phylogenomic classification of the fungal species.

**Results and discussion:**

Our results show that the CAZy content of these fungi does not correlate with their genome-based phylogeny, indicating that the CAZome has evolved separately from the overall genome, possibly under different selection pressures. In some cases, clear adaptation of the fungal genome to the host plant of the fungus can be observed, including an indication of parallel evolution among Sordariomycetes and *Dothidiomycetes* species. We mainly detected intergeneric variation in CAZome, while intrageneric diversity was low. This may indicate that initial adaptation is predominantly at the level of gene expression variation of specific CAZy genes, while over longer taxonomic timeframes, evolution of genome content becomes apparent.

## 1 Introduction

Plant pathogenic fungi have a significant impact on crop production and global food supply. They threaten global food security, causing major pre- and post-harvest crop losses, and affect all plant species on Earth (Savary et al., 2012).

While some plant pathogenic fungi are highly specific to a single plant species or group, others can cause diseases across a wide range of plants, demonstrating different abilities to adapt to the plant structure and defense mechanisms. These adaptations are closely related to the plant cell wall composition since the plant cell wall is the first physical barrier that fungi must overcome to cause infection. It is also a major source of carbon for fungi with its polysaccharide components (several hemicelluloses, cellulose, and pectin). The relative amounts and detailed structures of these polysaccharides differ per plant species and tissues (Benatti et al., 2012). Moreover, major differences in cell wall structure can be identified between monocots and dicots and between hardwood and softwood plant hosts (**Table 1**).

**Table 1:** Major polysaccharides in the cell walls of different terrestrial plants.

Plant type	Main cell wall polysaccharides	Minor cell wall polysaccharides
Monocots	Cellulose, arabinoxylan	
Dicots	Cellulose, pectin, xyloglucan	Xylan, (galacto-) mannan
Hardwood	Cellulose, glucuronoxylan	Galactomannan, pectin
Softwood	Cellulose, galactomannan	Glucuronoxylan, pectin

Fungi use carbohydrate active enzymes to degrade complex polysaccharides in the plant cell wall and biosynthesize or modify various carbohydrates. These enzymes have been catalogued in the Carbohydrate Active enzyme (CAZy) database (www.cazy.org) (Drula et al., 2022) in amino acid sequence-based families and subfamilies. This system allows an efficient evaluation of the genomic ability of a fungus to degrade complex substrates by analyzing the number of genes belonging to CAZy families associated with the degradation of a specific polysaccharide. Combining this analysis with growth profiling of plant biomass-based polysaccharides demonstrated that fungal genomes are often enriched in enzymes acting on the plant cell wall polysaccharides they typically encounter in their biotope. Two examples of this are the dung fungus *Podospora anserina* and the plant pathogenic fungus *Botrytis cinerea*. The genome of *P*. *anserina* has a strongly increased set of genes encoding cellulases and xylanases and a reduced set of pectinases, which matches the recalcitrant polysaccharides present in herbivore dung. It is supported by good growth of this species on xylan and cellulose and poor growth on pectin (Espagne et al., 2008). In contrast, *B*. *cinerea* shows good growth on pectin as well as an increased number of pectinase-encoding genes in its genome, matching the high pectin content in several of its host plants, such as tomato and strawberry (Amselem et al., 2011). The ability to degrade or alter specific carbon sources is therefore a beneficial trait for fungi, allowing them to compete and survive in their habitats. Therefore, fungal CAZy profiles provide information on the ecological capacity of fungi. They are particularly important in plant-associated fungi for establishing a link between their lifestyles and host ranges (Molina et al., 2024).

Most research on plant pathogenic fungi focuses on effector molecules and genes/proteins involved in or affecting the infection process, while fungal CAZy have been studied in terms of their defensive roles in fungal diseases in plants (Mafa et al., 2023) and habitat adaptation of fungi (Barrett et al., 2020; Baroncelli et al., 2024). These studies consisted of closely related plant pathogenic species with similar lifestyles across different hosts. However, there is limited data on whether phylogeny correlates with CAZy profiles at higher taxonomic levels in these fungi, and no definite answer on how to evaluate plant cell wall and fungal CAZy profiles together in plant pathogenic fungi. Studies have been performed within sets of *Dothideomycetes* (Ohm et al., 2012; Haridas et al., 2020) and sets of *Sordariomycetes* (Ledo Doval et al., 2025) which included comparisons of CAZy content of the respective genomes. However, no studies thus far evaluated whether species of these two fungal groups that infect the same host plants have similar CAZy profiles. In this study, we investigated the relationship between CAZy profiles of selected plant pathogenic *Dothideomycetes* and *Sordariomycetes* fungi, and related this to their taxonomic relationships as well as their host plant types.

## 2 Methodology

### 2.1 Selection of fungal genomes and phylogenetic analysis

In total, 98 fungal genomes of 56 species from *Dothideomycetes* and 42 species from *Sordariomycetes* were selected from the Joint Genome Institute (JGI) MycoCosm database (Grigoriev et al., 2014) based on the lifestyles (pathogens, latent pathogens, and endophytes) of the fungal species (**Supplementary Table 1**). Using the GenBank, USDA, and MycoCosm databases, reported host information has been recorded (last accessed October 10th, 2025). This information included the host plant name and plant type (dicot, monocot, hardwood, or softwood). All but three of the species have published genomes (see **Supplementary Table 1** for references), while for the other three, permissions were obtained from the data owners. The current names of the species were validated through the Index Fungorum and MycoBank databases.

Orthologous groups of 98 species were identified using OrthoFinder v2.5.5.2 (Emms and Kelly, 2019). A total of 165 single-copy orthologs were aligned independently using MAFFT v7.427 (Katoh and Standley, 2013) (option –maxiterate 1000). A maximum likelihood phylogeny from the concatenated amino acid alignments of the single-copy orthogroups was constructed with 1000 fast bootstrap replicates and selecting the best amino acid model for each gene partition using iqtree v3.0.0 (Wong et al., 2025) (options -m MFP -bb 1000 -safe). The phylogenetic tree was rooted to the midpoint and visualized using FigTree v1.4.4 (Girio et al., 2010).

The single-copy orthologs were identified in all genomes using OrthoFinder (Emms and Kelly, 2019). Reference protein sequences for each strain were obtained from the CAZy database (Drula et al., 2022) and aligned using Clustal Omega (Madeira et al., 2022).

### 2.2 CAZy annotation and statistical analysis

Gene copy data for the relevant CAZy were obtained from the JGI MycoCosm database (Emms and Kelly, 2019), which includes annotation of their CAZy content according to the CAZy annotation pipeline (Espagne et al., 2008), and exported via its Application Programming Interface (API). From these annotations, the numbers of genes were extracted in families that can be specifically assigned to degradation of either cellulose, xylan, mannan, xyloglucan, pectin, starch or inulin (**Supplementary Table 2**) based on previously published plant cell wall composition data (Vogel, 2008; Girio et al., 2010). Only families that could be assigned to specific polysaccharides were included in the analysis, while the families with activities against multiple polysaccharides (e.g., GH2, GH3) were excluded to allow a more specific comparison of the degradation potential for specific polysaccharides. In total, 59 CAZy families from four different CAZy groups (Auxiliary Activities (AA), n=3; Carbohydrate Esterases (CE), n= 3; Glycoside Hydrolases (GH), n=39; and Polysaccharide Lyases (PL), n=14) were included in the study (**Supplementary Table 1**). The CAZy families were divided into subfamilies and integrated into the dataset based on their substrates, except for the β-glucosidases of GH2. The β-glucosidases were split into extra- and intracellular enzymes using SignalP v5.0 (Nielsen et al., 1997).

Statistical comparison of CAZy content between different pathogen groups were performed with R (v 4.5.2) software. The p-value was calculated by non-parametric rank test using the default R function wilcox.test(). The principal component analysis was performed with R package “FactoMineR” and visualized with “ggplot2” package. Heatmap was drawn with R package “ComplexHeatmap”, using the default clustering parameters (“euclidean distance” and “complete clustering”).

## 3 Results

### 3.1 Differences in CAZome profiles are only partially based on taxonomic relationships of the fungal species

**Figure 1:**
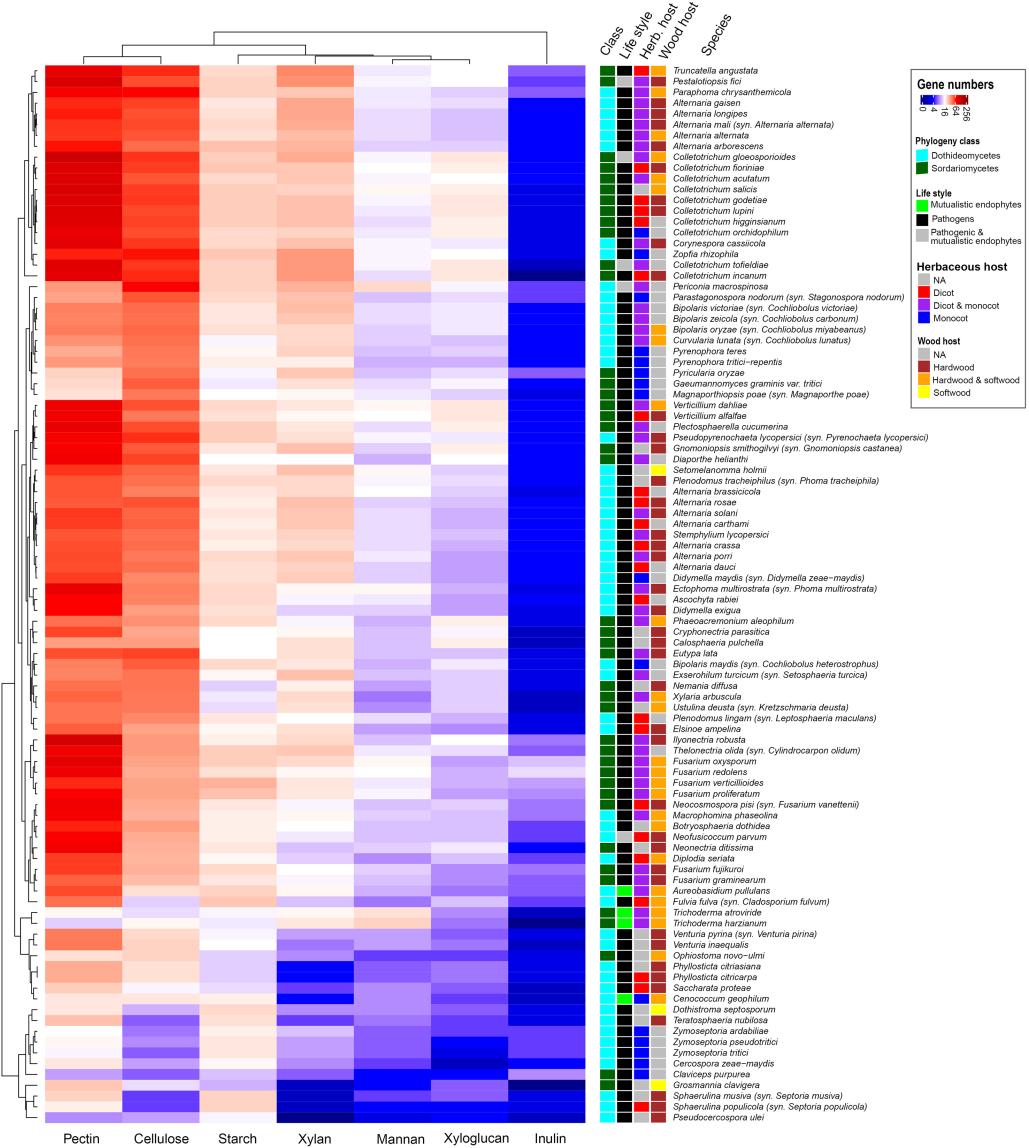
Clustered heatmap revealing similarity on overall CAZome content of fungal species related to the degradation of specific plant polysaccharides.

This study includes 56 *Dothideomycetes* (34 genera) and 42 *Sordariomycetes* (26 genera), which are mainly plant pathogenic fungi, but also four mutualistic endophytes and five species that can be found as both pathogen and mutualistic endophyte in different plants (**Supplementary Table 1**). The pathogens of both classes cover a wide range of host plants, including mono- and dicots, as well as hard- and softwoods (**Supplementary Table 1**). The clustered heatmap of the gene numbers related to the different polysaccharides revealed similarities between species from the two classes with similar host plants (**Figure 1**). This was particularly clear for a subgroup consisting of three *Sordariomycetes* (*Magnaporthiopsis poae*, *Pyricularia oryzae*, *Gaeumannomyces graminis*) and eight *Dothideomycetes* (*Pyrenophora tritici-repentis*, *Pyrenophora teres*, *Curvularia lunata*, *Bipolaris oryzae*, *Bipolaris zeicola*, *Bipolaris victoriae*, *Parastagonospora nodorum*, *Periconia macrospinosa*). All these species are associated with monocots and all show a strong reduction in putative pectinases (**Figure 1**), which matches the low pectin content of monocots.

**Figure 2:**
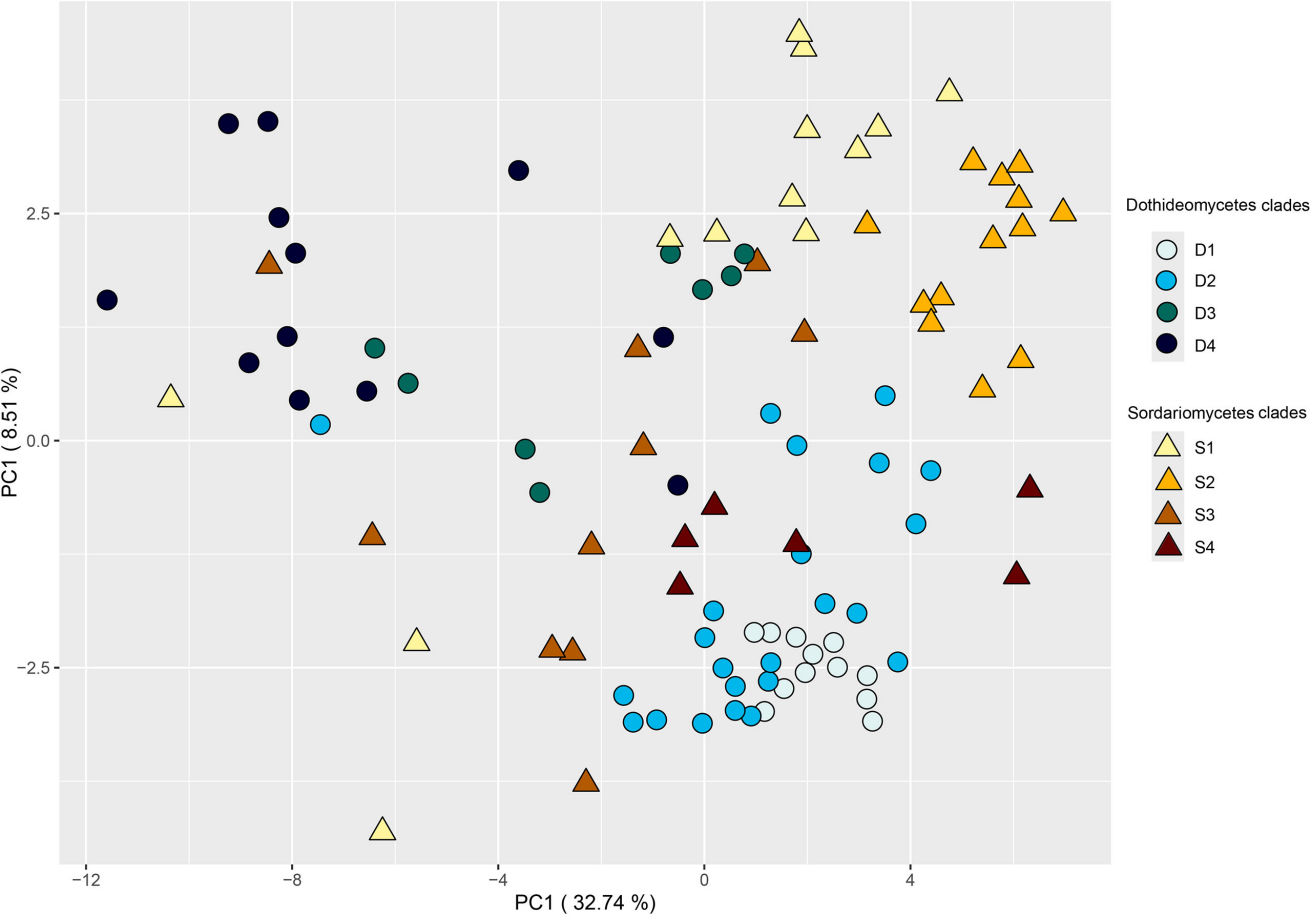
PCA plot of the CAZome content of the fungi of this study. The clades refer to the branches identified in the genome-based phylogenetic tree (**Supplementary Figure 1**).

When the number of genes relating to degradation of plant polysaccharides was plotted on the genome-based phylogenetic tree (**Supplementary Figure 1**), this revealed that in most cases, closely related species had similar CAZy profiles. However, a large diversity, in terms of variation in gene copy numbers, was observed in both fungal classes, also within the four main branches of the genome-based phylogenetic tree of each class (denoted as D1–D4 and S1–S4 in **Supplementary Figure 1**). This already indicates that the CAZome of these species has evolved separately from the overall genome. A principal component analysis (PCA) of the CAZome content revealed differences in species diversity of these branches (**Figure 2**). Species from branches D1 and S2 clustered closely, while species from the other branches showed a more diverse distribution. This is likely due to D1 consisting only of *Alternaria* species and S2 mainly of *Colletotrichum* species, while the other branches contain a broader range of genera. It therefore indicates relatively low diversity of the CAZome within a genus, while the diversity between genera (even if they are closely related) is much higher.

### 3.2 Host plant adaptation in CAZome content is apparent when evaluating specific CAZy families

Analysis of the variation within specific CAZy families confirms the low diversity within several genera (e.g., *Alternaria*, *Bipolaris, Colletotrichum*, and *Fusarium*), as species from these genera cluster together in a heatmap based on the number of genes per individual CAZy family (**Supplementary Figure 2**). According to the data presented in **Supplementary Figure 2**, **Supplementary Table 1**, not all genera with low CAZy diversity revealed a clear link between low CAZome diversity and specific host categories. For example, *Bipolaris maydis* is associated only with monocots, whereas all other *Bipolaris* species in our dataset were reported from multiple host types; yet these fungi clustered together based on their CAZome content. On the other hand, in *Fusarium*, *F*. *fujikuroi* and *F*. *graminearum* have been reported from monocots, dicots, and softwoods, and they clustered separately from the other *Fusarium* species, which have been reported from all four host plant types. In *Alternaria*, the species related to three or more host types, except *A*. *solani* and *A*. *porri*, clustered separately from other *Alternaria* species related to one or two host types (**Supplementary Figure 2**). Similar to the clustering based on overall genes involved in a specific polysaccharide, no taxonomic distinction was visible when the clustering was based on the individual CAZy families. This demonstrates that similar CAZy content was present in both *Dothideomycetes* and *Sordariomycetes*, suggesting an evolutionary development for CAZy genes that is different from the genome as a whole.

**Figure 3:**
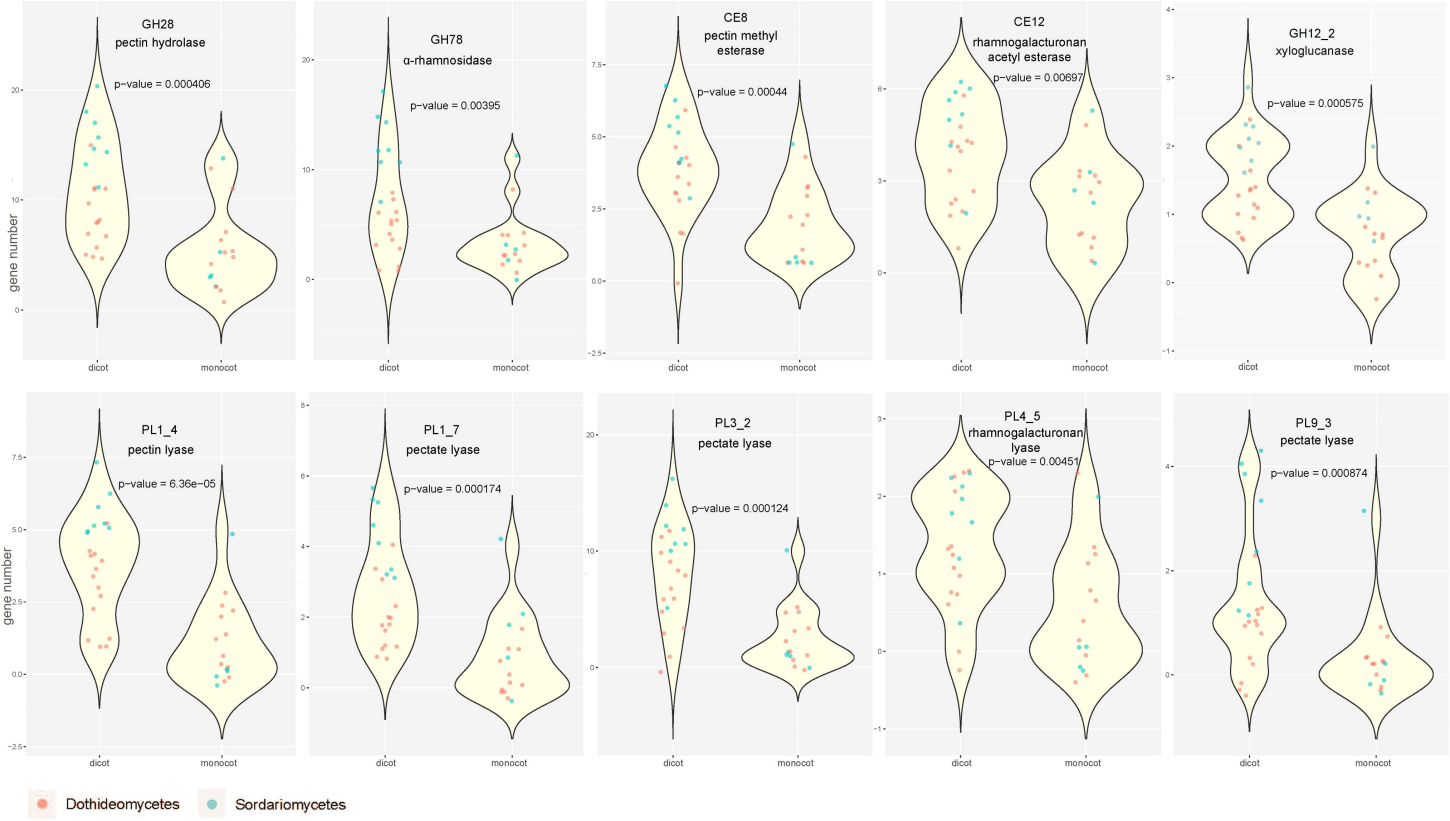
Violin plots comparing the number of genes in 10 pectin-related CAZy families between monocot and dicot pathogens. The p-values were calculated with Wilcoxon rank test based on genes in pathogens uniquely attacking herbaceous monocot and dicot plants.

This pattern becomes even more apparent when the number of genes is compared between fungi that have monocots or dicots, respectively, as host plants. A major difference in the cell wall composition of monocots and dicots is the presence of pectin, which is zero to low in monocots, while it is high in most dicots. When the number of genes in pectin-related CAZy families was compared between dicot and monocot-specific plant pathogens (see **Supplementary Table 1** for details) using violin plots (**Figure 3**), a clear increase in pectinolytic genes was observed for dicot pathogens for most pectin-specific CAZy families. More detailed comparison showed that most of these pectinolytic genes expanded in dicot pathogens of both Sordariomycete and Dothideomycetes, except that GH78 and PL4_5 only significantly (P-value < 0.05) increased in Sordariomycete (**Supplementary Figure 3**).

**Figure 4:**
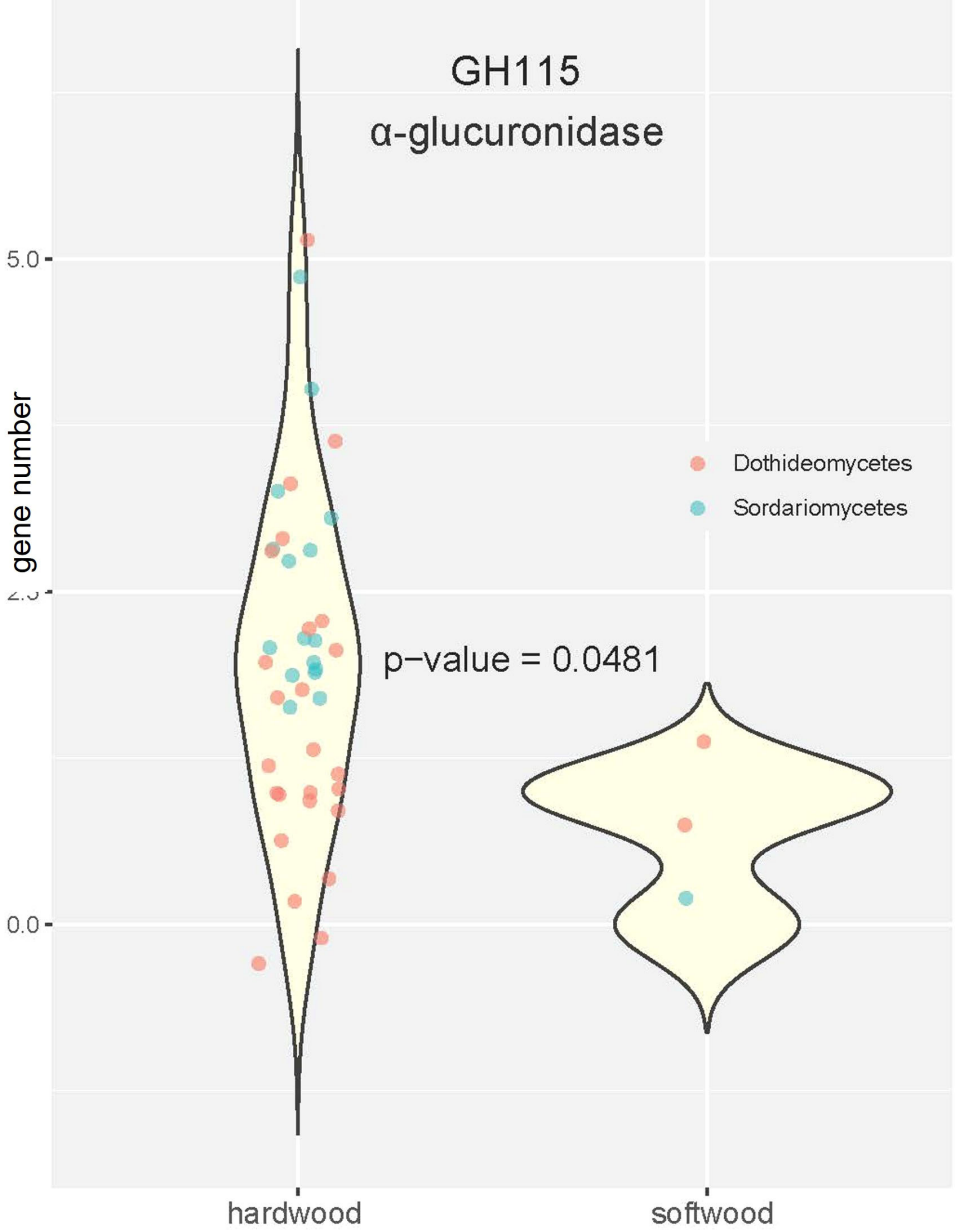
Violin plots comparing the number of genes in the α-glucuronidase containing GH115 family. The p-value was calculated with Wilcoxon rank test based on copies of GH115 genes in pathogens uniquely attacking softwood and hardwood.

In contrast, no clear differences in pathogens of softwood or hardwood could be found, with the exception of CAZy family GH115 (**Figure 4**). This family contains α-glucuronidases that remove (4-O-methyl)-D-glucuronoyl residues from xylan. The main hemicellulose in hardwood is glucuronoxylan, while it is galactomannan in softwood (Vogel, 2008), suggesting that the expansion of this family could be an adaptation to the cell wall composition of hardwood pathogens. However, there may be a possible bias in our analysis caused by small size of softwood pathogens in our statistical comparison. Therefore, the detailed function of α-glucuronidases for hardwood pathogens requires further examination.

### 3.3 A broad host plant range does not consistently result in a larger CAZy gene set

In the current study, *Dothideomycetes* were associated with 85 plant species, whereas *Sordariomycetes* were reported from 82 plant species. Among those, 48 were common in two classes (**Supplementary Table 1**). Higher variability in host plant preference did in general not correlate with higher CAZy numbers in the genomes of these species. Only *Fulvia fulva* and *Macrophomina phaseolina* had a higher total number of CAZy copies and broader host ranges than other taxa in *Dothideomycetes* (**Figure 1**; **Supplementary Table 1**). This link was not detected across other fungal groups and in some cases; species that are associated with fewer plant types had more CAZy gene copies (e.g., among *Alternaria*, *Bipolaris*, and *Fusarium* species) (**Figure 1**; **Supplementary Table 1**). Interestingly, some monocot and wood associated fungi had an overall reduction in CAZy genes (**Figure 1**, bottom clade). This could indicate that these species use other wood components as a carbon source or that they produce very high levels of the few enzymes they possess. The latter strategy is well described for *Trichoderma reesei*, which is a very efficient cellulose degrader despite having a modest set of cellulolytic genes in its genome (Martinez et al., 2008). It contrasts to other good cellulose degraders, such as *Podospora anserina*, which possesses a highly expanded set of cellulolytic genes (Espagne et al., 2008).

## 4 Discussion

In this study, we investigated whether the CAZome composition of a set of *Dothideomycetes* and *Sordariomycetes* plant pathogens correlates with the polysaccharide composition of the cell wall of their host plants. The plant cell wall is a physical barrier that pathogens need to overcome, and its composition differs significantly in different plant types (Vogel, 2008; Benatti et al., 2012). Therefore, it is conceivable that adaptation of fungi to their host plant would include genomic evolution towards a set of enzymes tailored towards the prevalent polysaccharides in the host cell wall. This would then suggest that distantly related fungi with the same host plant would have more similar CAZomes than closely related fungi with different host plants. Alternatively, if the evolution of these species did not include adaptive alterations in their CAZome, the similarity in their CAZome would decrease with increasing taxonomic distance. Our study demonstrated that CAZomes evolve partially based on adaptation, as clustering of the fungal species based on their CAZy gene composition (**Figures 1**, **2**; **Supplementary Figure 2**) does not match their genome-based phylogeny (**Supplementary Figure 1**). In fact, in several cases *Dothideomycetes* and *Sordariomycetes* fungi cluster together, despite their taxonomic distance. Previous studies comparing 18 (Ohm et al., 2012) and 101 (Haridas et al., 2020) *Dothideomycetes* also indicated that, within this fungal class, the differences in CAZome composition did not correlate with the taxonomic distance of the species, and suggested adaptation to life style (saprobe or pathogen) and host plant (for pathogens). Similarly, a study comparing nine *Sordariomycetes* fungi showed no correlation between the difference in CAZome and their taxonomic distance (Ledo Doval et al., 2025).

Interestingly, this absence of correlation is mainly observed at the genus level, while species within the genus have largely similar CAZomes, suggesting that these adaptations are mostly not recent. In our study, we saw close clustering of multiple species of *Zymoseptoria* (n=3;clade D4), *Sphaerulina* (n=2; clade D4), *Phyllosticta* (n=2; clade D3), *Trichoderma* (n=2; clade S1), *Venturia* (n=2; clade D3), *Fusarium* (n=6; clade S1), *Verticillium* (n=2; clade S2), *Pyrenophora* (n=2; clade D2), *Magnaporthiopsis*/*Pyricularia* (n=2; clade S3), *Bipolaris* (n=3; clade D2), *Colletotrichum* (n=10; clade S2) and *Alternaria* (n=11; clade D1) based on the number of genes in individual CAZy families (**Supplementary Figure 2**). This similarity is maintained when the clustering was based on the number of genes related to a specific polysaccharide (**Figure 1**), except for *Alternaria*, where the species are split into two groups of seven and four species each.

Similarity of CAZomes within fungal genera has been reported before for *Colletotrichum* (Baroncelli et al., 2024), but differential gene expression of CAZy genes suggested that the adaptation within the genus may be largely at the post-genomic level. This is consistent with studies in the genus *Aspergillus* where the CAZome similarity was high within each section (Vesth et al., 2018; Theobald et al., 2024; Nybo et al., 2025; Theobald et al., 2025), with more variation between the sections, likely due to the taxonomic breadth of this genus. Functional studies demonstrated that within each section, variation between the species could be observed in secreted protein profiles (Meijer et al., 2011; Benoit et al., 2015; Mäkelä et al., 2018), suggesting that also in this genus, recent adaptations are predominantly at the level of gene expression rather than in genome content.

However, the diversity in CAZomes can only be partially linked to the host plant cell wall composition. The clustering of subsets of *Sordariomycetes* and *Dothideomycetes* species based on their CAZome suggests parallel evolution in species of these two fungal classes, but only in some cases the clustered species attack a similar host plant. The clearest example of parallel evolution of CAZomes was observed in three *Sordariomycetes* (*Pyricularia oryzae*, *Gaeumannomyces graminis* var. *tritici*, and *Magnoporthiopsis poae*) and two *Dothideomycetes* (*Pyrenophora teres* and *P*. *tritici*-*repentis*) monocot pathogens that all showed a significant reduction of pectin-related CAZy genes (**Figure 1**). We should however consider that many of the species included in our study can attack multiple hosts, often including both monocots and dicots (**Supplementary Table 1**), widening the range of cell wall polysaccharides they would be expected to degrade. In addition, as mentioned above, genome content is only part of the story. Adaptations also occur at the post-genomic level and especially if species have more recently expanded or reduced their host plant range, this may be more at the level of gene expression profiles than genome content.

In our dataset, some hemibiotrophic fungi (e.g., *Colletotrichum* species) showed CAZy content similar to or higher than that of most necrotrophic fungi, such as *Fusarium*, *Alternaria*, and *Curvularia* species. One necrotrophic fungus, *Grosmannia clavigera*, had the lowest CAZy content. This indicates that the functional relevance of CAZome may be species-dependent rather than solely attributable to the lifestyle of the species.

The results show that taxonomically distant fungi that infect similar host plants can have similar CAZy profiles, indicating that these enzyme sets may have evolved independently during the evolution of these fungi. Therefore, sharing a host can lead to the presence of similar CAZy families across different fungi. However, in some taxa, regardless of their phylogenetic closeness, the CAZy profile showed differences even though they infect the same plant types. This suggests that these fungi may target different components of the cell wall and/or use different enzymes to degrade the same plant polysaccharide.

## 5 Conclusions

We evaluated the evolution of the CAZome of a large set of *Dothideomycetes* and *Sordariomycetes* plant pathogenic fungi. The absence of correlation between the clustering of the species based on their CAZome and their genome-based phylogeny indicated that the CAZy content of these species has evolved separately from their overall genome. Overall comparison of the *Dothideomycetes* and *Sordariomycetes* species used in this study suggests that the evolution of the CAZy content followed both lineage-specific and plant-host-specific evolutionary patterns.

It should be noted that the results of this study indicate the “potential” of the selected plant pathogenic fungi for degradation of plant polysaccharides and have not yet taken into account the expression of these genes during plant infection. Gene expression can vary significantly, even among closely related species, as previously reported for *Aspergillus* and *Colletotrichum* (De Vries et al., 2017; Mäkelä et al., 2018; Baroncelli et al., 2024), and appears to be the initial adaptation of the species in their polysaccharide degradation approach. Future studies into the transcriptomic profile of a large set of plant pathogenic fungi during plant infection would provide additional insights into species-specific adaptations in cell wall degradation.

## Data Availability

The original contributions presented in the study are included in the article/**Supplementary Material**. Further inquiries can be directed to the corresponding author.
