# Supplementary material for: CAZome comparison in relation to host plant for selected Sordariomycete and Dothidiomycete plant pathogenic fungi
Source: Front Fungal Biol. 2026 Mar 10;7:1789997. doi: 10.3389/ffunb.2026.1789997 (PMC13008963; doi:10.3389/ffunb.2026.1789997)
Supplement: Supplementary file 1 [file DataSheet1.pdf]

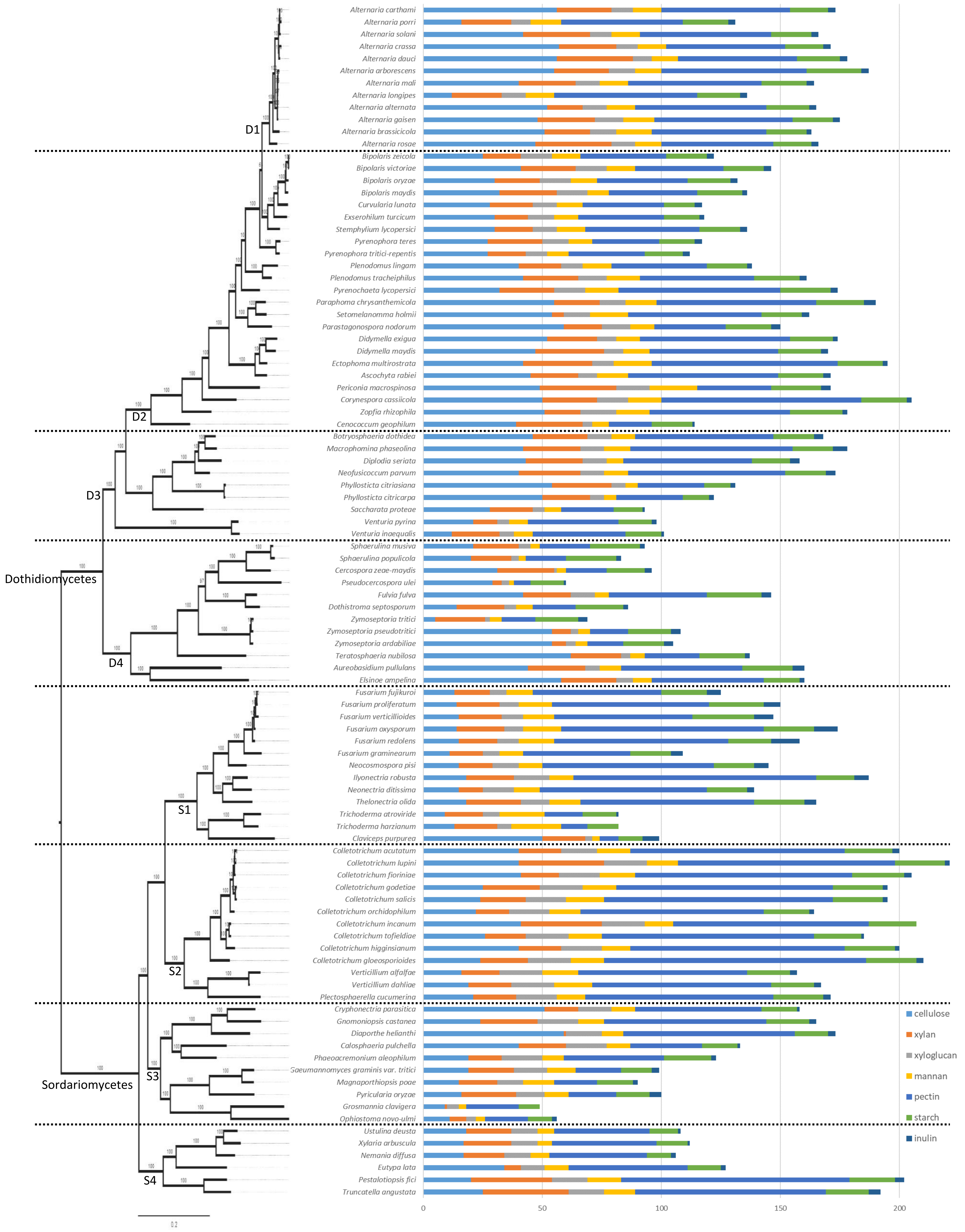

Supplemental Figure S1. Number of genes putatively involved in the degradation of specific plant polysaccharides plotted on the genome-based taxonomic tree.
