# Supplementary material for: CAZome comparison in relation to host plant for selected Sordariomycete and Dothidiomycete plant pathogenic fungi
Source: Front Fungal Biol. 2026 Mar 10;7:1789997. doi: 10.3389/ffunb.2026.1789997 (PMC13008963; doi:10.3389/ffunb.2026.1789997)
Supplement: Supplementary file 3 [file DataSheet3.pdf]

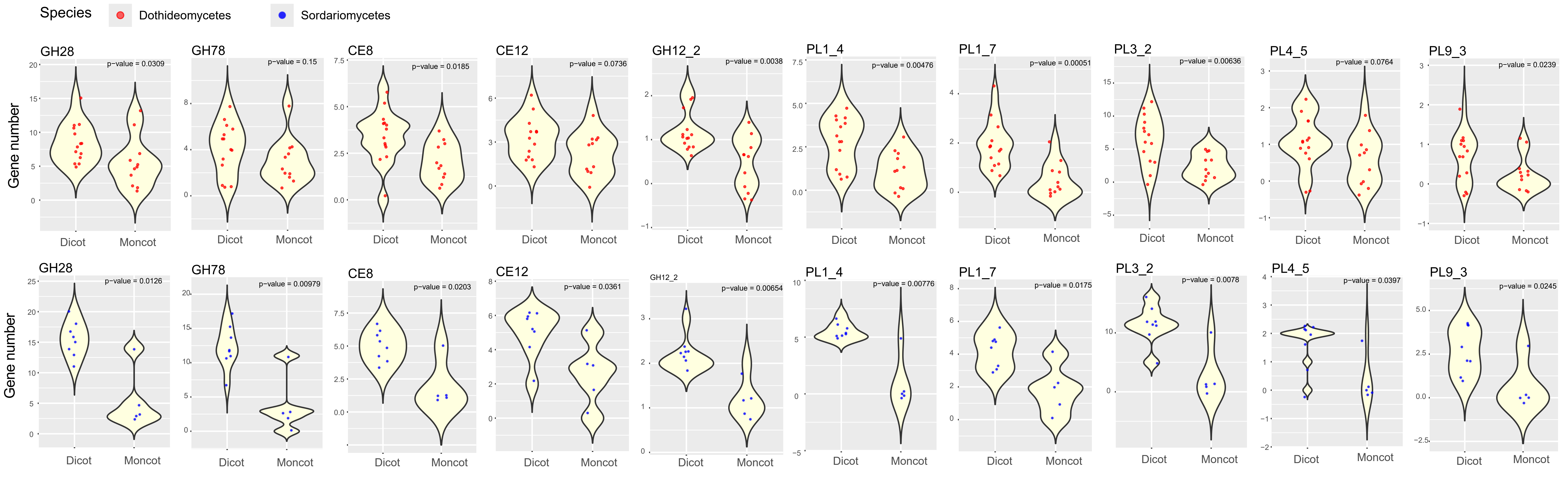

Supplemental Figure S3. Violin plots comparing the number of genes in 10 pectin-related CAZy families between monocot and dicot pathogens in Dothideomycetes and Sordariomycetes. The p-values were calculated with Wilcoxon rank test based on genes in pathogens uniquely attacking herbaceous monocot and dicot plants.
